# Pre-Reduction-Activated Carbon-Based Zinc Vanadate Nanosheet Composite with Zn–O–V Performance Synergy for Machine Learning-Enabled Multiplex Pesticide Detection

**DOI:** 10.3390/nano16171082

**Published:** 2026-08-31

**Authors:** Lihua Zhong, Bingrui Zou, Xin Li, Shuiju Guo, Haijun Guan, Chou Mo, Qianfeng Wang, Yuchao Wang, Hongyu Wang, Xin Kou, Yongpeng Zhao, Hui Huang

**Affiliations:** 1 College of Mechanical and Electrical Engineering, Sichuan Agricultural University, Ya’an 625000, China; 2 College of Water Conservancy and Hydropower Engineering, Sichuan Agricultural University, Ya’an 625000, China; 3 Party School of CPC Wuwei Municipal Committee (Wuwei Administrative College), Wuwei 733000, China; 4 College of Mechanical Engineering, Qinghai University, Xining 810016, China; 5 College of Resources, Sichuan Agricultural University, Chengdu 610000, China

**Keywords:** electrochemical sensing, machine learning, pesticides, zinc vanadate, differential pulse voltammetry

## Abstract

The simultaneous and accurate detection of multiple pesticide residues remains a critical challenge in electrochemical sensing. Herein, a strategy is proposed for the in situ growth of interconnected Zn_3_(OH)_2_V_2_O_7_·2H_2_O (ZVO) nanosheets on carbon cloth (CC), forming ZVO/CC electrodes for the simultaneous detection of thiophanate-methyl and diuron. A negative-potential pre-reduction treatment is employed to regulate the interfacial electronic structure and activate sensing sites of ZVO/CC electrodes. During pre-reduction, partial V^5+^ is reduced to V^4+^, accompanied by the formation of oxygen vacancies, which reconstruct local electronic states and decrease charge-transfer resistance. Meanwhile, the chemically integrated Zn–O–V framework exhibits a performance synergy, resulting in significantly enhanced and well-distinguished electrochemical responses toward the target pesticides. The ZVO/CC electrode achieves linear detection ranges of 0.1–25 μM for thiophanate-methyl and 0.1–40 μM for diuron, with low detection limits of 12.4 nM and 28.5 nM, respectively. Furthermore, machine learning algorithms are introduced to resolve partial overlapping signals, enabling simultaneous pesticide classification and concentration prediction. The integration of interfacial engineering with machine learning provides an effective strategy for achieving simultaneous multi-pesticide detection at the nanomolar level.

## 1. Introduction

The extensive and combined use of persistent pesticides, such as thiophanate-methyl and diuron, in modern agriculture leads to their long-term accumulation in the environment and agricultural products, posing potential risks to the nervous, endocrine, hepatic, and renal systems [1,2,3]. The frequent coexistence of multiple pesticides further increases the complexity of their combined toxicological effects, many of which remain poorly understood [4,5]. Therefore, rapid and sensitive methods for simultaneous multi-component detection are highly needed for food safety and environmental monitoring [6,7,8]. At present, pesticide residue analysis primarily relies on conventional techniques such as high-performance liquid chromatography (HPLC) and gas chromatography–mass spectrometry (GC–MS) [9,10,11]. Electrochemical sensing is attractive for rapid, portable, and potentially on-site pesticide analysis. However, the detection limits and linear ranges achieved in simultaneous multi-pesticide sensing depend on the target analytes, electrode materials, sensor configuration, degree of signal overlap, and sample matrix, rather than being intrinsic advantages of the electrochemical method [12,13,14,15]. Therefore, the rational design of electrode structures and regulation of interfacial electronic states are key to improving analytical performance [16,17,18,19].

Various electrode engineering approaches have been explored to improve pesticide detection. Hybrid interfaces based on carbon materials and metal oxides exhibit superior sensing performance. For example, Gong et al. prepared a ZrO_2_-modified graphene nanosheet electrode via a solvothermal method, which achieved a detection limit of 2.28 nM for methyl parathion within a linear range of 0.008–3.42 μM [20]. Beyond compositional regulation, optimization of the electrode microstructure can increase the accessible surface area and enhance electrochemical activity. For example, a cobalt–zinc phosphide/CoO/ZnO core–shell heterostructure enabled the simultaneous electrochemical detection of carbofuran and diuron, with detection limits of 2.98 and 2.52 nM, respectively, over a linear range of 2–185 μM [21]. However, simultaneous detection of multiple pesticides remains challenging due to overlapping redox signals and matrix interference from real samples, which hinder selective identification on a single electrode [22,23]. Defect engineering provides an effective strategy for regulating the electronic structure of metal oxides and constructing multi-site synergistic interfaces. In particular, the introduction of oxygen vacancies via metal co-doping enables modulation of adsorption energy and enhances electrochemical activity [24].

Recently, layered double hydroxide (LDH) materials have attracted considerable attention in electrochemical sensing because of their layered structures and abundant surface hydroxyl groups. These features favor interfacial adsorption and electron transfer, showing strong potential for multi-component detection [25]. For example, a Mg–Al LDH/poly-m-amino-benzenesulfonic acid composite electrode has been employed for the simultaneous detection of methyl parathion and fenitrothion [26]. Inspired by this strategy, layered materials with multiple active sites are expected to generate more distinguishable electrochemical responses in multi-component systems. Zn_3_(OH)_2_V_2_O_7_·2H_2_O (ZVO) is a hydrated zinc pyrovanadate with a porous framework composed of Zn–O octahedral layers connected by V_2_O_7_ pyrovanadate groups, with water molecules accommodated in the framework cavities [27]. The hydroxyl groups and crystal water in ZVO can improve surface hydrophilicity and facilitate electrolyte penetration, ion transport, and interfacial reactions. Moreover, the multivalent redox chemistry of vanadium provides electroactive sites [28], whereas Zn-containing sites may contribute to the adsorption and local enrichment of pesticide molecules, as suggested by the reported adsorptive extraction of thiophanate-methyl using chitosan–ZnO nanoparticles, the surface-dependent adsorption of diuron on ZnO, and the enhanced electrochemical oxidation response of diuron at a ZnO-modified electrode [29,30,31]. These complementary functions may enable molecular adsorption and interfacial electron transfer to proceed within the chemically integrated Zn–O–V framework. However, pristine ZVO is dominated by V^5+^ species and thus lacks sufficient electrochemically active sites. Moreover, its intrinsically low conductivity results in inefficient charge transfer. Regulating the vanadium valence states and introducing oxygen vacancies via a pre-reduction strategy can effectively generate abundant active sites and improve interfacial charge-transfer kinetics for multi-target detection. In addition, the direct growth of ZVO on conductive carbon cloth (CC) establishes close electrical contact between ZVO and the conductive substrate, shortens the electron transport pathways, avoids the use of insulating binders, and enhances structural stability [32].

Previous metal oxide/carbon-cloth sensing platforms have mainly exploited the flexible conductive substrate and high-surface-area oxide architectures for signal enhancement. For example, ZnO nanostructures directly grown on carbon cloth have been employed for electrochemical pesticide detection [33], while vanadate-based sensors such as DyVO_4_ and DyVO_4_/graphene oxide have also been reported for fenitrothion and carbendazim sensing [34,35]. These representative studies primarily focused on the intrinsic electrocatalytic properties, morphology, or conductive-composite effects of the sensing materials. In contrast, the present ZVO/CC system introduces a negative-potential pre-reduction step before pesticide detection. This treatment induces partial V^5+^→V^4+^ conversion and increases oxygen-vacancy-related defect centers, providing an additional tunable activation process beyond the as-prepared electrode structure. Meanwhile, the increasing signal complexity in multi-pesticide systems necessitates the integration of machine learning to enable accurate signal discrimination and multi-analyte detection [14,36]. Herein, a pre-reduction-activated ZVO/CC electrode is constructed for simultaneous detection of multiple pesticides. The in situ grown ZVO features interconnected nanosheets, which facilitate electrolyte penetration and mass transport. Its layered architecture exposes abundant Zn–O–V interfacial sites, enabling efficient electron transfer. Upon pre-reduction, partial V^5+^ is converted into lower-valence vanadium species, accompanied by the generation of oxygen vacancies. These changes modulate the local electronic structure, create more electroactive centers, and enhance the interaction between the electrode surface and pesticide molecules. Furthermore, machine learning is introduced to extract multidimensional features from electrochemical signals, enabling both pesticide classification and concentration prediction. Thus, the present system integrates pre-reduction-induced defect/valence regulation, Zn–O–V performance synergy, simultaneous dual-pesticide sensing, and machine-learning-assisted signal analysis within a single sensing platform.

## 2. Experimental Section

### 2.1. Chemicals and Materials

CC was purchased from CeTech Co., Ltd. (Taichung, Taiwan, China). Zinc nitrate hexahydrate (Zn(NO_3_)_2_·6H_2_O), ammonium metavanadate (NH_4_VO_3_), sulfuric acid (H_2_SO_4_), nitric acid (HNO_3_), sodium hydroxide (NaOH), acetone, and absolute ethanol were of analytical grade and obtained from Macklin Biochemical Co., Ltd. (Shanghai, China). Potassium dihydrogen phosphate (KH_2_PO_4_), dipotassium hydrogen phosphate (K_2_HPO_4_), potassium ferricyanide (K_3_[Fe(CN)_6_]), potassium ferrocyanide (K_4_[Fe(CN)_6_]), potassium chloride (KCl), and sodium chloride (NaCl) were obtained from the same supplier. Deionized (DI) water was used throughout all experiments.

### 2.2. Preparation of ZVO/CC Electrode

To improve the hydrophilicity and interfacial adhesion of CC and promote the uniform growth of active materials, the CC was pretreated by cleaning and acid oxidation. The CC was cut into 2 × 2 cm^2^ pieces and sequentially ultrasonicated in acetone, absolute ethanol, and DI water for 15 min each, followed by drying. The cleaned CC was then immersed in a mixed solution of H_2_SO_4_, HNO_3_, and DI water (1:1:1 by volume) and treated at 140 °C for 12 h [37,38]. This treatment introduced oxygen-containing functional groups onto the CC surface. After naturally cooling to room temperature, the acid-treated CC was rinsed with DI water until neutral pH and dried at 60 °C.

ZVO was grown in situ on the pretreated CC through a one-step hydrothermal method. Briefly, 1.18 mmol of Zn(NO_3_)_2_·6H_2_O and 3.54 mmol of NH_4_VO_3_ were dissolved in 10 mL of DI water under stirring. Next, 0.2 g of NaOH (5.0 mmol) was added, and the solution was stirred for another 30 min. The precursor solution was transferred into a Teflon-lined stainless steel autoclave containing a piece of pretreated CC and maintained at 180 °C for 12 h [39]. After cooling to room temperature, the samples were collected, rinsed several times with DI water, and dried to obtain the ZVO/CC electrodes. For comparison, V_2_O_5_/CC, ZnO/CC, and bare CC electrodes were also prepared under the same hydrothermal conditions.

### 2.3. Characterization

The morphology and microstructure of the samples were characterized using field-emission scanning electron microscopy (SEM, ZEM18) and transmission electron microscopy (TEM, JEM-2100). Crystal structures were analyzed by X-ray diffraction (XRD, D/max 2550). Surface elemental composition and chemical valence states were examined by X-ray photoelectron spectroscopy (XPS, Nexsa G2). Chemical bonding structures were characterized using Fourier-transform infrared spectroscopy (FTIR, Nicolet iS5) and Raman spectroscopy (LabRAM HR Evolution). Surface wettability was evaluated by contact angle measurements using a contact angle goniometer (JC2000D1).

### 2.4. Electrochemical Measurements

All electrochemical measurements, including cyclic voltammetry (CV), electrochemical impedance spectroscopy (EIS), and differential pulse voltammetry (DPV), were performed on a CHI 760E electrochemical workstation (Shanghai Chenhua Instrument Co., Ltd., Shanghai, China) using a three-electrode system. ZVO/CC was used as the working electrode, while a platinum plate and an Ag/AgCl (saturated KCl) electrode served as the counter and reference electrodes, respectively. CV and EIS measurements were carried out in a solution containing 5.0 mM K_3_[Fe(CN)_6_]/K_4_[Fe(CN)_6_] and 0.1 M KCl to evaluate electrochemical behavior and interfacial charge transfer. DPV measurements were performed in phosphate-buffered saline (PBS) for the detection of thiophanate-methyl and diuron. For the pre-reduced ZVO/CC group, the electrode was first scanned from −1.0 to 0 V in blank PBS to complete the pre-reduction process. The target pesticides were then added to the PBS, followed by DPV measurement over the analytical potential range of 0 to 1.4 V. For the control group without pre-reduction, the pesticides were added directly to the PBS, and the DPV measurement was conducted from 0 to 1.4 V under otherwise identical experimental conditions.

## 3. Results and Discussion

**Figure 1:**
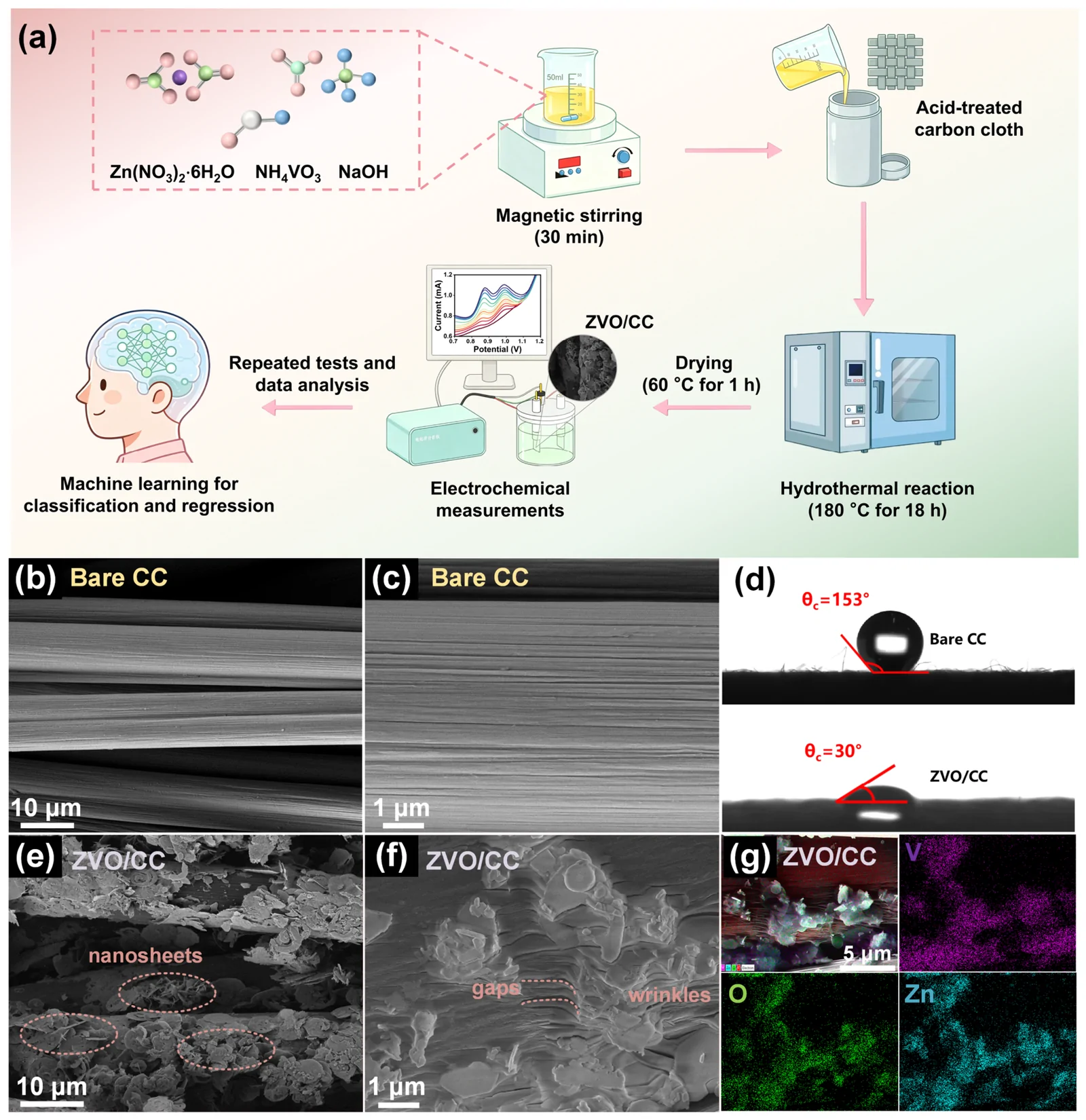
(**a**) Schematic illustration of the overall research workflow, including electrode preparation, electrochemical detection, and machine learning analysis. (**b**,**c**) SEM images of bare CC. (**d**) Comparison of water contact angles between bare CC and ZVO/CC. (**e**,**f**) SEM images of ZVO/CC. (**g**) EDS elemental mapping of C, O, V, and Zn in ZVO/CC.

The performance of electrochemical sensing electrodes strongly depends on abundant active sites and good surface wettability, which collectively facilitate analyte diffusion and interfacial charge transfer [40]. Based on this principle, a ZVO/CC film electrode with an interconnected nanosheet architecture is rationally designed. The overall research strategy and electrode fabrication process are presented in Figure 1a. Briefly, ZVO nanosheets were first grown on the acid-treated CC through a hydrothermal method to construct the ZVO/CC electrode. Subsequently, the ZVO/CC electrode was further optimized by tuning the precursor concentration and Zn/V molar ratio and then employed for pesticide detection. Finally, machine learning models were developed based on data from multiple measurements to enable pesticide classification and concentration prediction.

In contrast to the relatively smooth morphology of bare CC (Figure 1b,c), the ZVO/CC electrode (Figure 1e,f) features a dense network of interconnected ZVO nanosheets fully covering the CC surface. The nanosheets are approximately 200 nm thick and extend laterally to several micrometers, with numerous wrinkles distributed across their surfaces. The interwoven nanosheets generate abundant irregular pores, most of which range from several hundred nanometers to approximately 2 μm in diameter. These pores increase the accessible surface area and expose more electrochemically active sites, thereby facilitating interfacial electrochemical reactions. Meanwhile, the interconnected channels provide efficient pathways for electrolyte ion diffusion and accelerate charge transfer, resulting in an enhanced electrochemical response [41]. Water contact angle measurements (Figure 1d) demonstrate the improved wettability of ZVO/CC. The contact angle decreases from 153° for bare CC to 30° after ZVO growth, indicating a substantial improvement in hydrophilicity. This improvement promotes electrolyte infiltration and increases the accessibility of active sites, which benefits interfacial charge transfer. Furthermore, EDS elemental mapping results (Figure 1g) confirm the uniform distribution of Zn, V, and O elements across the CC surface. These results verify the successful and homogeneous growth of ZVO nanosheets on the CC substrate.

**Figure 2:**
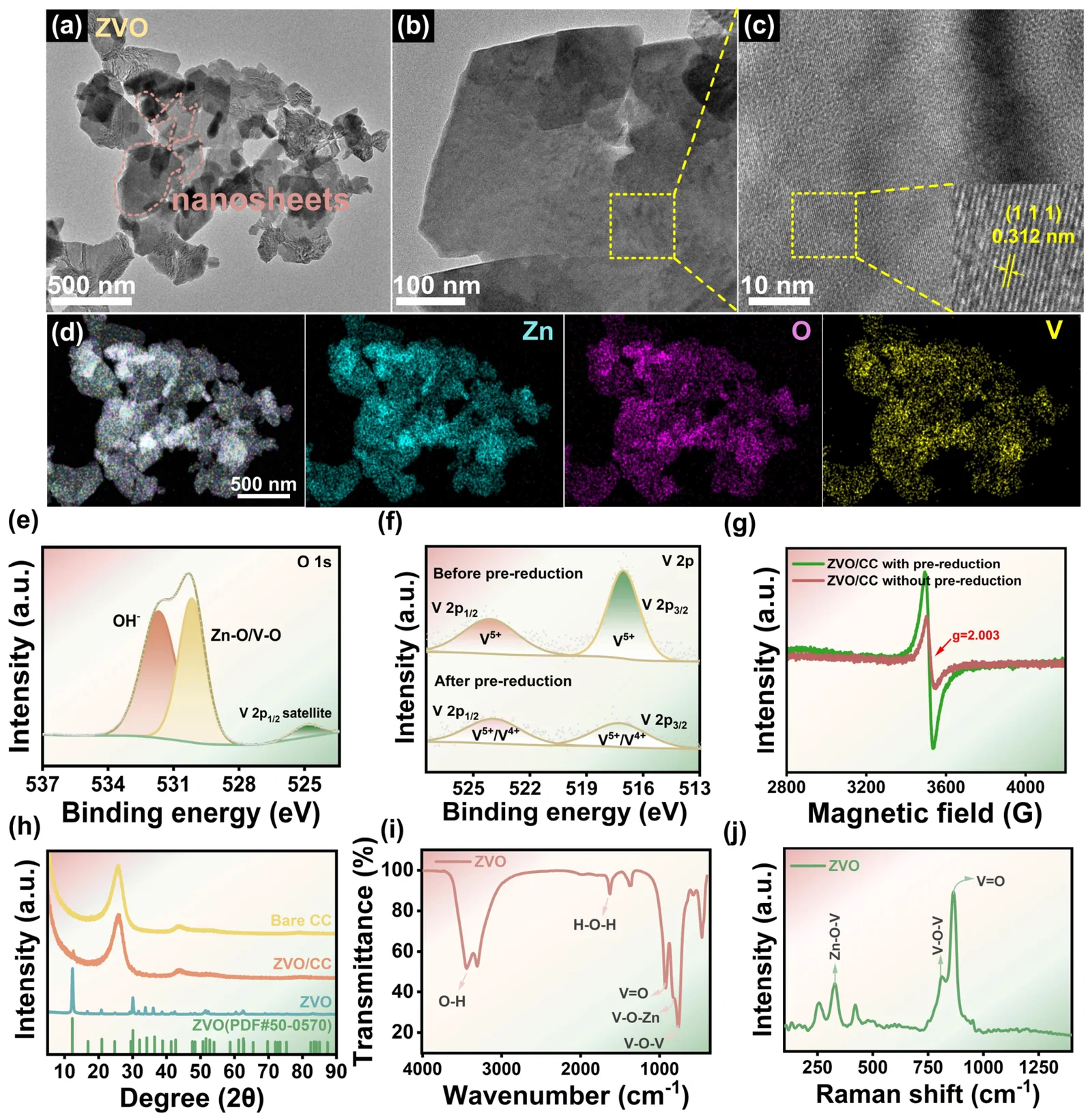
(**a**–**c**) TEM images of ZVO. (**d**) EDS elemental mapping of ZVO. High-resolution XPS spectra of (**e**) O 1s and (**f**) V 2p of ZVO/CC before and after pre-reduction. (**g**) EPR spectra of ZVO/CC with and without pre-reduction. (**h**) XRD patterns of bare CC, ZVO/CC, and ZVO powder. (**i**) FTIR spectrum and (**j**) Raman spectrum of ZVO.

To further investigate the microstructure and crystal structure of ZVO, TEM characterization was performed. Figure 2a–c show the typical sheet-like morphology of ZVO. Lattice fringe images (Figure S1) show an interplanar spacing of approximately 0.312 nm, corresponding to the (111) crystal plane. EDS indicates that Zn, V, and O elements are uniformly distributed across the material without obvious aggregation or phase separation, demonstrating the good compositional homogeneity of ZVO (Figure 2d). Based on the uniform elemental distribution of the ZVO sample, the surface chemical states of the constituent elements were further analyzed by XPS. As shown in Figure S2, the survey spectrum confirms the presence of Zn, V, and O in the ZVO sample. In the high-resolution O 1s spectrum (Figure 2e), the peaks at approximately 530.1 and 531.5 eV are assigned to lattice oxygen and hydroxyl oxygen, respectively. To investigate the changes induced by negative-potential pre-reduction, the high-resolution V 2p spectra of ZVO/CC before and after pre-reduction were compared. As shown in Figure 2f, the V^5+^-associated signal decreased after pre-reduction, accompanied by a slight shift of the V 2p peak envelope toward lower binding energy, indicating an increased contribution from lower-valence vanadium species and supporting partial V^5+^-to-V^4+^ conversion during the pre-reduction process. In addition, the pre-reduced ZVO/CC exhibited a markedly enhanced EPR signal compared with pristine ZVO/CC (Figure 2g). Under identical acquisition conditions and after normalization to sample mass, the double-integrated EPR intensity increases from 1.00 for pristine ZVO/CC to 2.46 for the pre-reduced sample, indicating an increased population of paramagnetic defect centers associated with oxygen deficiency. Oxygen-vacancy-related defects in oxides can exhibit different electronic states depending on the number and localization of trapped electrons [42]. In a reducible vanadium-containing oxide, the electrons associated with oxygen deficiency may remain localized at the vacancy or be transferred to neighboring V^5+^ centers, reducing them to V^4+^. The latter process provides a possible local charge-compensation mechanism and may lead to vacancy–V^4+^ defect complexes. Therefore, the appearance of lower-valence vanadium species together with the enhanced EPR signal is consistent with coupled oxygen-deficiency/V^4+^ defect chemistry. These results confirm that redox-active V species and surface oxygen-containing groups have been formed in the system, leading to the construction of abundant electrochemically active sites and a Zn–O–V synergistic electronic structure. This configuration can effectively enhance interfacial charge-transfer kinetics and accelerate reversible redox reactions, thereby improving the overall electrochemical sensing performance.

The phase composition and crystallinity of bare CC, ZVO/CC, and pure ZVO powder were further characterized by XRD. As shown in Figure 2h, the diffraction patterns of ZVO/CC match well with those of pure ZVO powder, with no detectable impurity peaks, confirming the successful formation of highly crystalline ZVO on the CC substrate. FTIR and Raman spectroscopy were further employed to probe the chemical bonding structure of ZVO. In the FTIR (Figure 2i), the broad band at around 3440 cm^−1^ and the peak at 1625 cm^−1^ are associated with hydroxyl groups and interlayer water molecules. The characteristic peaks at 923 and 763 cm^−1^ correspond to V=O and V–O–V vibrations, confirming the formation of the zinc vanadate framework and abundant oxygen-containing functional structures. Consistently, the Raman spectrum (Figure 2j) shows the characteristic peaks near 878 cm^−1^ and 965 cm^−1^, assigned to the asymmetric stretching vibration of V–O–V and the V=O stretching vibration, respectively. These results collectively confirm the well-defined layered structure and robust framework of ZVO, which are expected to provide structural stability and abundant redox-active sites, thereby facilitating efficient charge transfer in electrochemical sensing.

**Figure 3:**
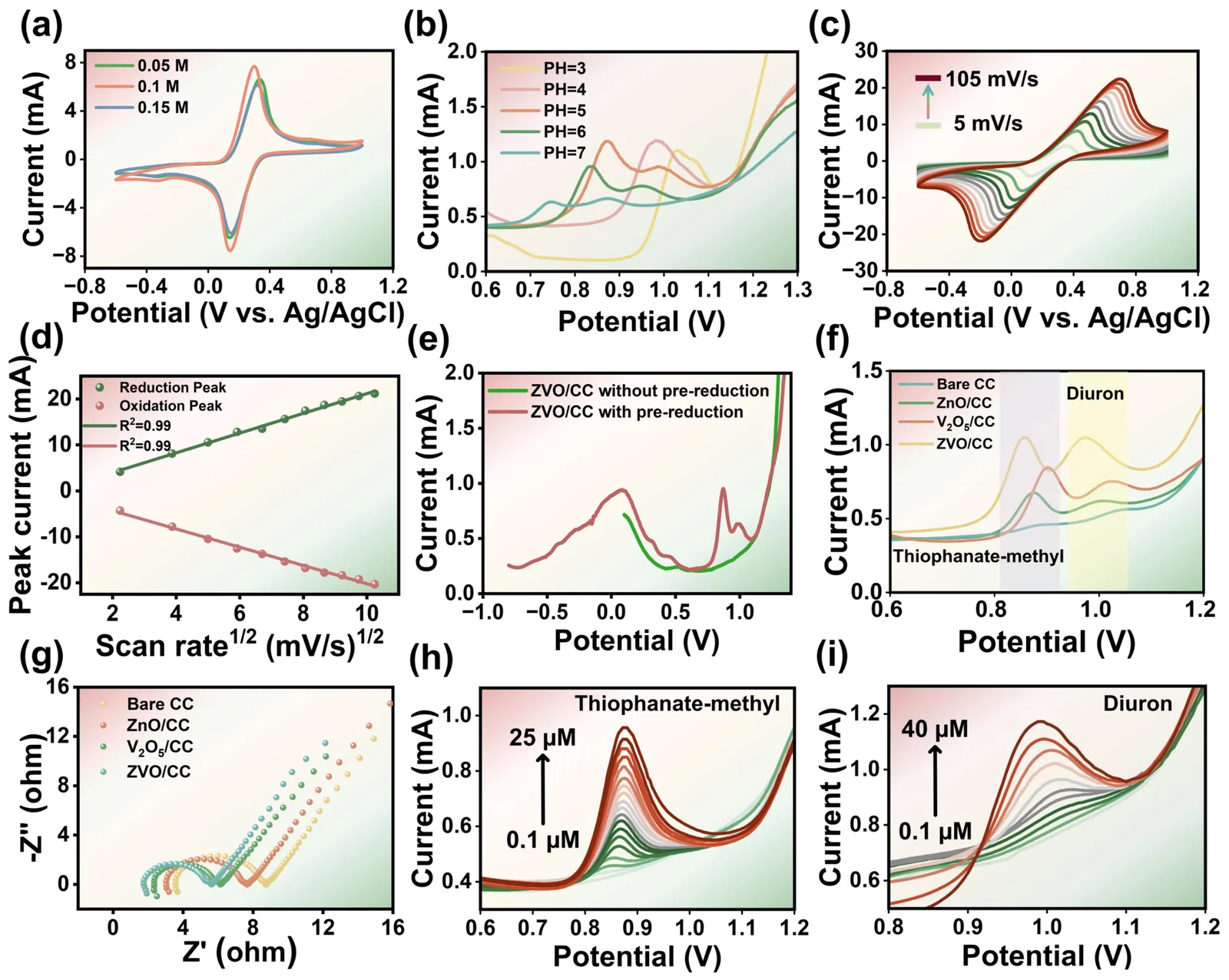
(**a**) CV curves under different precursor concentrations. (**b**) Influence of buffer solution pH on the current response. (**c**) CV curves of ZVO/CC at various scan rates. (**d**) Linear relationship between redox peak currents and the square root of the scan rate. (**e**) Comparison of electrochemical responses toward thiophanate-methyl and diuron with and without pre-reduction treatment. (**f**) Comparison of electrochemical sensing toward thiophanate-methyl and diuron for bare CC, ZnO/CC, V_2_O_5_/CC, and ZVO/CC. (**g**) Nyquist plots of bare CC, ZnO/CC, V_2_O_5_/CC, and ZVO/CC. (**h**) DPV response curves with successive additions of thiophanate-methyl. (**i**) DPV response curves with successive additions of diuron.

To achieve optimal electrochemical performance, the electrode preparation and testing conditions were systematically optimized. Figure 3a shows the CV responses of electrodes prepared from ZVO precursor solutions with different concentrations, while Figure S3 shows the CV response results obtained at different Zn:V molar ratios in the precursor. The ZVO/CC electrode fabricated with a precursor concentration of 0.1 M and a Zn:V ratio of 1:3 exhibits the highest redox peak current and was therefore selected for subsequent electrochemical measurements. The effect of PBS buffer pH on the current response of ZVO/CC was further investigated (Figure 3b). The peak current reaches a maximum at pH 5.0, indicating that a weakly acidic environment is favorable for proton-assisted electrochemical processes and facilitates the diffusion of target molecules [38]. Figure 3c presents the CV curves of ZVO/CC at scan rates from 5 to 105 mV/s. The peak currents increase linearly with the square root of the scan rate (Figure 3d, R^2^ = 0.99), indicating a diffusion-controlled process [43]. This diffusion-dominated behavior facilitates efficient mass transport of analytes to the electrode surface, thereby contributing to enhanced electrochemical sensing performance.

Subsequently, DPV measurements of thiophanate-methyl and diuron were performed using ZVO/CC electrodes with and without negative-potential pre-reduction. For the control group without pre-reduction, the DPV measurement was initiated at 0 V. For the pre-reduced group, the ZVO/CC electrode first underwent a negative-potential pre-reduction process from −1.0 to 0 V, followed immediately by measurement over the same positive-potential detection range under otherwise identical experimental conditions. As shown in Figure 3e, ZVO/CC without pre-reduction exhibited no discernible oxidation peaks for either pesticide, whereas the pre-reduced ZVO/CC electrode displayed distinct oxidation peaks with substantially enhanced peak currents. This behavior is associated with the partial reduction of V^5+^ to lower-valence vanadium species and the concurrent increase in oxygen-vacancy-related defect centers. The V^4+^/V^5+^ redox chemistry can strongly influence the electrochemical activity of vanadium oxides, while oxygen-vacancy engineering can modify surface adsorption, electronic structure, and charge-transfer behavior in electrochemical sensing systems [19,44,45]. The DPV responses of different electrodes toward the two pesticides are compared in Figure 3f. ZVO/CC exhibits the highest response currents for both analytes at the same concentration. To investigate the synergistic interaction between Zn and V, ZnO/CC and V_2_O_5_/CC control electrodes were prepared for comparison. In addition, a physically mixed ZnO+V_2_O_5_/CC electrode was prepared to distinguish the contribution of the chemically integrated Zn–O–V structure from the simple coexistence of ZnO and V_2_O_5_. The physically mixed electrode exhibited only weak responses toward both pesticides, with oxidation peak currents markedly lower than those of ZVO/CC (Figure S4). EIS was conducted to evaluate the interfacial charge transfer behavior. The Nyquist plot of ZVO/CC exhibits the smallest semicircle diameter in the high-frequency region in Figure 3g, indicating the lowest charge-transfer resistance value of 3.378 Ω (Table S1). The larger straight-line slope of ZVO/CC in the low-frequency region further suggests faster ion diffusion. These results demonstrate a clear performance synergy of the chemically integrated ZVO structure compared with the individual ZnO and V_2_O_5_ components and their physical mixture. The key spectroscopic and electrochemical parameters associated with pre-reduction, including the V 2p spectral evolution, normalized EPR response, charge-transfer resistance, and pesticide response currents, are summarized in Table S2.

The electrochemical simultaneous sensing capability of the ZVO/CC electrode toward thiophanate-methyl and diuron was further evaluated. Figure 3h shows the DPV responses of ZVO/CC toward thiophanate-methyl over the concentration range of 0.1–21.5 μM. The oxidation peak current at approximately +0.86 V increases progressively with concentration while maintaining a stable peak profile. Figure 3i shows the corresponding DPV responses toward diuron over the concentration range of 0.1–40 μM, with the characteristic oxidation peak appearing at approximately +1.0 V. The calibration equations are I = 0.02416C + 0.46976 (R^2^ = 0.99) for thiophanate-methyl and I = 0.01054C + 0.83217 (R^2^ = 0.99) for diuron, where I is the peak current (mA) and C is the pesticide concentration (μM). Using LOD = 3σ/S, the LODs are calculated to be 12.4 nM for thiophanate-methyl and 28.5 nM for diuron (Figure S5). These results indicate that ZVO/CC possesses high sensitivity toward single-pesticide detection.

**Figure 4:**
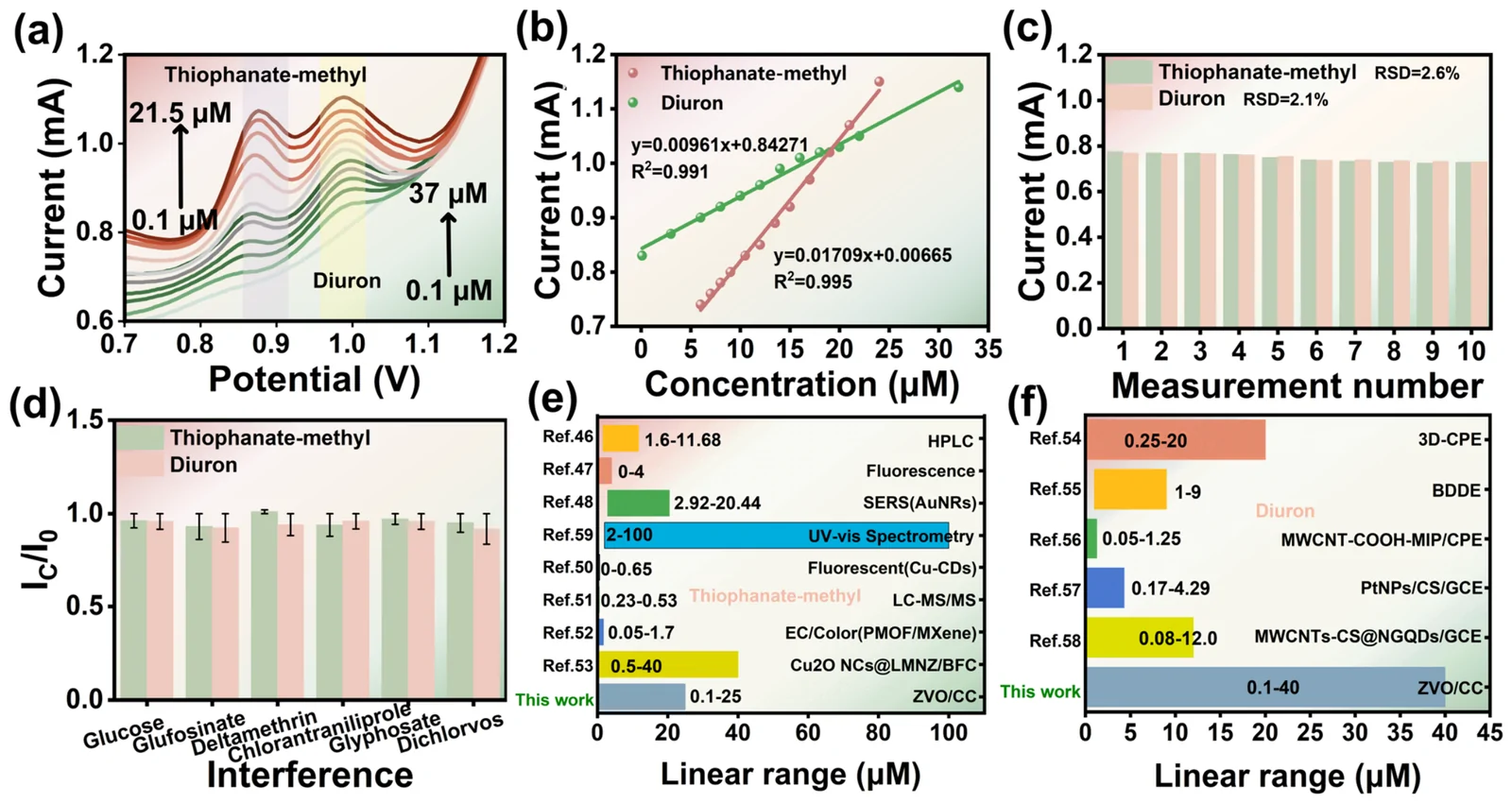
(**a**) DPV curves for the simultaneous detection of thiophanate-methyl and diuron with successive additions. (**b**) Linear regression analysis of current responses versus concentrations in the dual-component system. (**c**) Repeatability test based on current responses for 10 consecutive measurements. (**d**) Selectivity assessment based on current responses in the presence of various interfering species. Comparison of detection performance for (**e**) thiophanate-methyl [46,47,48,49,50,51,52,53] and (**f**) diuron with previously reported methods and electrodes [54,55,56,57,58].

The simultaneous detection capability of the electrode was further investigated. Figure 4a shows the DPV responses of thiophanate-methyl and diuron in a mixed solution. Two oxidation peaks are clearly observed, and their peak potentials are consistent with those obtained in the single-component tests. Although slight peak overlap exists, no obvious interference is observed. As the concentrations of both pesticides increase simultaneously, the corresponding peak currents increase independently without obvious signal suppression or peak shifts. Figure 4b shows the calibration curves for simultaneous detection. The R^2^ values of both pesticides remain above 0.99, demonstrating the capability of ZVO/CC for simultaneous quantitative detection in mixed systems. Figure 4c evaluates the short-term operational stability and repeatability of ZVO/CC under sensing conditions through 10 consecutive DPV measurements. The relative standard deviations (RSDs) of the peak currents are all below 3%, indicating excellent sensing stability. In addition, five independently prepared ZVO/CC electrodes fabricated using the same procedure exhibited consistent responses toward thiophanate-methyl and diuron, with RSDs of 0.41% and 0.63%, respectively, demonstrating good electrode-to-electrode reproducibility (Figure S6). Long-term storage stability was evaluated separately. After storage in a sealed environment at room temperature for 60 days, the ZVO/CC electrode retained 95.3% and 99.8% of its initial peak-current responses toward thiophanate-methyl and diuron, respectively (Figure S7). Furthermore, the selectivity of the ZVO/CC electrode was evaluated against six representative interferents, including glucose, glufosinate, deltamethrin, chlorantraniliprole, glyphosate, and dichlorvos. In the interference test, the concentrations of thiophanate-methyl and diuron were each fixed at 20 μM, while each interferent was tested at 100 μM, corresponding to a fivefold excess relative to the target analytes. As shown in Figure 4d, the peak-current ratio (I_c_/I_0_) remained close to 1 after the addition of these interferents, indicating limited interference from the specifically tested species under the investigated conditions. Additional DPV measurements shown in Figure S8 independently confirmed that the characteristic oxidation responses of thiophanate-methyl and diuron remained distinguishable in the presence of the tested interferents. These results indicate satisfactory selectivity against the specifically examined interferents under the present experimental conditions. This anti-interference behavior can be attributed to the stronger interfacial interactions between the O- and N-containing functional groups of the target pesticides and the hydroxylated Zn–O–V surface, together with the differences between the oxidation potentials of the target pesticides and those of the interfering species. Consequently, the interfering pesticides show limited competition for the active sites and produce negligible currents at the characteristic oxidation potentials of the target analytes.

Figure 4e,f and Tables S3 and S4 compare the performance of the ZVO/CC electrode with previously reported electrochemical electrodes and other detection methods [46,47,48,49,50,51,52,53,54,55,56,57,58]. The ZVO/CC electrode exhibits lower detection limits and wider linear ranges, or at least comparable sensing performance. Notably, compared with electrochemical electrodes such as MWCNT-COOH-MIP/CPE, the ZVO/CC electrode demonstrates superior overall sensing performance. This performance mainly originates from the unique interconnected nanosheet and interfacial structure of ZVO/CC. The layered structure provides Zn and V bimetallic active centers. During pre-reduction, electrochemically active V^4+^ species and oxygen vacancies are generated on the surface. These species act as the primary active sites for pesticide oxidation. In addition, the interconnected nanosheet structure facilitates the rapid transport of reactants and products. As a result, the electrode exhibits high sensitivity and reliable multi-component detection capability.

To evaluate the practical applicability of the sensing system, spike–recovery tests were performed in tap water and strawberry matrices [59]. Briefly, 100.0 g of homogenized fresh strawberry sample was mixed with 30 mL of acetonitrile and thoroughly stirred for 2 min. The upper homogenate was then collected and centrifuged at 4000 rpm for 5 min. The resulting supernatant was diluted with PBS to obtain the test solution. Thiophanate-methyl and diuron were spiked into the test solution at different concentration levels for recovery evaluation. DPV measurements were then conducted using the ZVO/CC electrode. As summarized in Table 1, the recoveries for both pesticides range from 97.40% to 101.35%, with RSD below 3%. These results indicate satisfactory recovery and precision for thiophanate-methyl and diuron determination in the investigated spiked tap-water and strawberry matrices.

**Figure 5:**
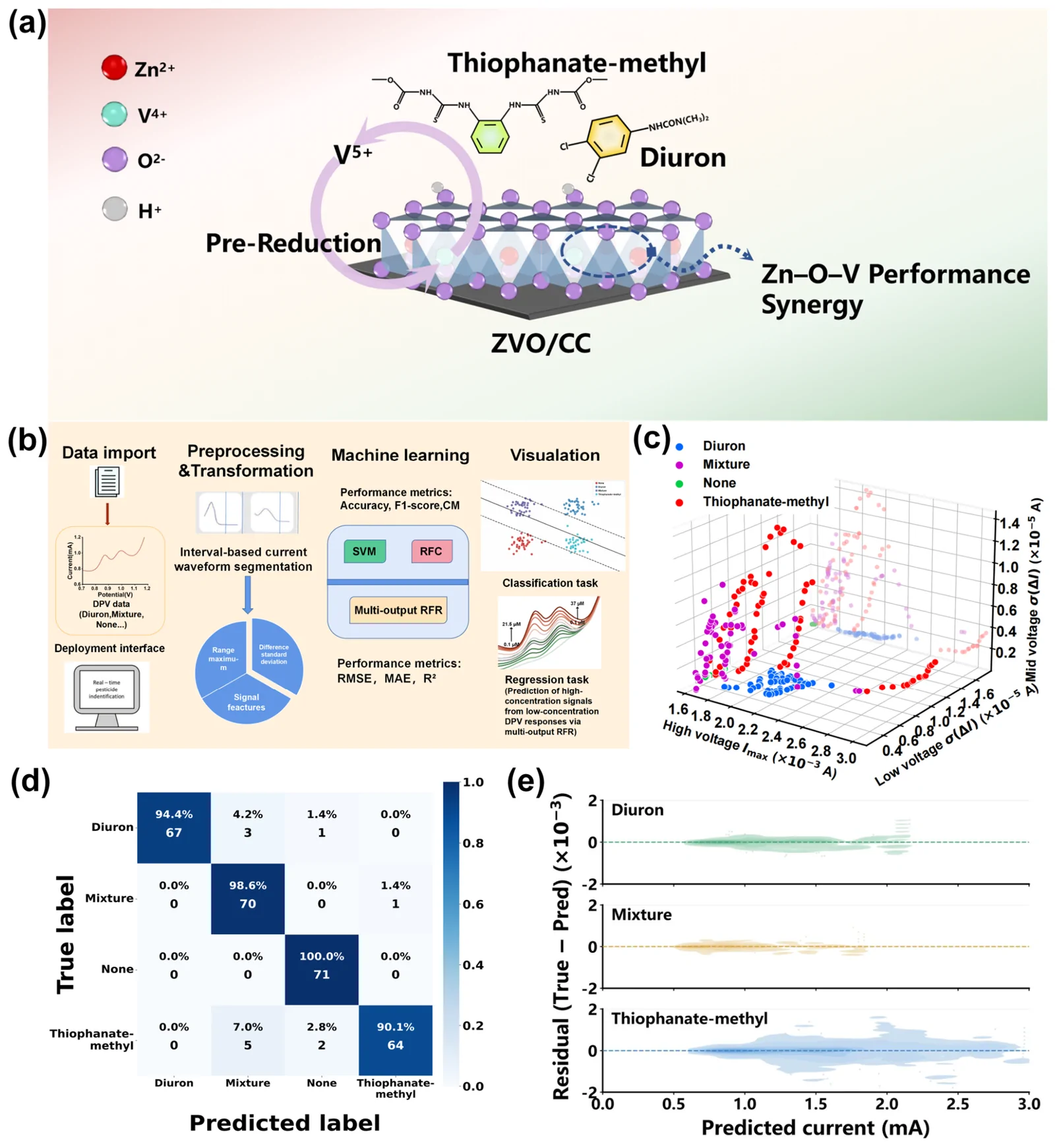
(**a**) Schematic illustration of the electrochemical sensing mechanism involving multi-site synergy induced by pre-reduction. (**b**) Schematic representation of the overall machine learning workflow. (**c**) The 3D scatter plot of the extracted electrochemical features. (**d**) Confusion matrix of the classification model results. (**e**) Residual analysis plot of the regression model.

These findings suggest that the sensing response of ZVO/CC arises from a synergistic interfacial process involving multiple active sites rather than a single active center. As shown in Figure 5a, the chemically integrated Zn–O–V structure exhibits a clear performance synergy in pesticide sensing. In this configuration, Zn sites preferentially adsorb target molecules through coordination with N- and O-containing groups, while low-valence V^4+^ species provide efficient electron transfer pathways. Meanwhile, oxygen vacancies modify the local electronic structure and strengthen the interaction between the analytes and the electrode surface.

Although this synergistic effect improves the multi-component sensing capability of ZVO/CC, partial signal overlap still exists in the binary system, making conventional univariate calibration insufficient for accurate analysis of mixed samples. Therefore, a machine learning strategy was introduced for signal classification and concentration prediction (Figure 5b). After baseline correction and normalization of all DPV curves, the onset current, peak current, and peak potential were extracted to construct a three-dimensional feature space (Figure 5c). Samples from different categories exhibit a certain degree of clustering in this space. However, partial overlap persists, indicating that linear discrimination alone is insufficient for reliable classification.

A support vector machine (SVM) model with a radial basis function (RBF) kernel was used for signal classification [60]. A total of 284 electrochemical measurements (71 measurements for each category) were included in the classification dataset. The measurements were acquired using five independently fabricated ZVO/CC electrodes. Within each replicate series, measurements sharing the same replicate index were obtained using the same electrode and the same independently prepared 100 mL PBS solution during cumulative pesticide addition. Under the original classification protocol, the dataset was divided into training and test subsets at a ratio of 4:1. Input features were standardized using StandardScaler. Candidate C values of 2, 3, 4, and 5 were examined during model development, and the final SVM implementation used C=3, γ = “scale”, and random_state = 1. The classification results obtained on the test subset (Figure 5d and Figure S9) showed an overall accuracy of 96%. The recall values for the background (None) and mixed samples (Mixture) were 1.00 and 0.99, respectively. The precision and F1-scores of the two single-pesticide categories were both above 0.94, while the macro-average and weighted-average F1-scores were both 0.96. To further assess the influence of group-level data partitioning, an additional group-aware robustness analysis was performed by keeping measurements from the same electrode/solution cumulative-addition sequence within the same group. The grouped analysis yielded an accuracy of 90.3 ± 5.5% and a macro-averaged F1-score of 86.0 ± 6.3%. These results demonstrate satisfactory classification performance under both the original and the more stringent group-aware validation settings.

A random forest (RF) model was further employed for concentration prediction [61]. In the signal-domain analysis corresponding to Figure 5e, the RF model was used to describe the relationship between pesticide concentration and the electrochemical current response. Figure 5e presents the display-scaled residuals between the measured and RF-predicted current responses, providing a visual assessment of prediction bias. To further evaluate quantitative concentration prediction, an additional concentration-level RF analysis was performed using the electrochemical response features as inputs and pesticide concentration as the prediction target. The concentration-prediction performance was evaluated on a group-held-out electrode/solution sequence using R^2^, RMSE, and MAE, and the results are summarized in Figure S10. For the single-analyte systems, the test R^2^ values were 0.966 and 0.949 for thiophanate-methyl and diuron, respectively, with RMSE values of 2.027 and 2.235 μM and MAE values of 1.419 and 1.554 μM. For the mixed-analyte system, the corresponding test R^2^ values were 0.900 and 0.895, with RMSE values of 1.253 and 1.861 μM and MAE values of 0.832 and 1.234 μM, respectively. These results demonstrate satisfactory quantitative prediction performance for both single-analyte and mixed-analyte samples under the present group-held-out evaluation.

## 4. Conclusions

In this work, ZVO nanosheets were grown in situ on CC substrates via a hydrothermal method and subsequently activated by negative-potential pre-reduction for the simultaneous electrochemical detection of thiophanate-methyl and diuron. The interconnected nanosheet framework of ZVO promotes electrolyte penetration and charge transport while exposing abundant Zn–O–V interfacial sites. The pre-reduction process induces the formation of low-valence vanadium species and oxygen vacancies, thereby reconstructing the interfacial electronic structure and accelerating charge-transfer kinetics. Benefiting from the performance synergy of the chemically integrated Zn–O–V structure, the ZVO/CC electrode enables the simultaneous detection of thiophanate-methyl and diuron over linear ranges of 0.1–25 μM and 0.1–40 μM, with detection limits of 12.4 and 28.5 nM, respectively. Furthermore, machine learning-assisted analysis enables simultaneous pesticide classification and concentration prediction. This work provides a promising strategy for efficient multi-pesticide detection through the synergistic integration of multi-active sites and data-driven analysis.

## Figures and Tables

**Table 1:** Spike–recovery results for thiophanate-methyl and diuron in tap-water and strawberry matrices.

Sample	Analyte	Added (μM)	Found (μM)	Recovery (%)
Tap water	Thiophanate-methyl	10	9.82	98.20
		20	20.27	101.35
	Diuron	10	9.74	97.40
		35	35.36	101.03
Strawberry	Thiophanate-methyl	10	9.79	97.90
		20	19.55	97.75
	Diuron	10	9.96	99.60
		35	35.38	101.09

## Data Availability

The original contributions presented in this study are included in the article/Supplementary Material. Further inquiries can be directed to the corresponding authors.

## Supplementary Materials

The following supporting information can be downloaded at https://www.mdpi.com/article/10.3390/nano16171082/s1. Figure S1: Interplanar spacing analysis of ZVO. Figure S2: (a) XPS survey spectrum of ZVO/CC. (b) High-resolution C 1s spectrum. Figure S3: CV curves of ZVO/CC prepared with different precursor Zn:V ratios. Figure S4: Electrochemical sensing toward thiophanate-methyl and diuron for ZnO+V_2_O_5_/CC. Figure S5: Linear relationships between analyte concentration and oxidation peak current for (a) thiophanate-methyl and (b) diuron, respectively. thiophanate-methyl 14 points, diuron 10 points, n = 3 per concentration, blank n = 5. Figure S6: Electrode-to-electrode reproducibility of independently prepared ZVO/CC electrodes for the detection of thiophanate-methyl and diuron. Figure S7: Long-term storage stability of the ZVO/CC electrode toward thiophanate-methyl and diuron after sealed storage at room temperature for 60 days. Figure S8: DPV responses of the ZVO/CC electrode toward thiophanate-methyl and diuron before and after the addition of fivefold concentrations of (a) glucose, (b) glufosinate, (c) deltamethrin, (d) chlorantraniliprole, (e) glyphosate, and (f) dichlorvos. Figure S9: Class-wise and overall performance metrics of the SVM classifier for pesticide discrimination. Figure S10: Quantitative performance metrics of the random-forest concentration-prediction model for thiophanate-methyl and diuron under single-analyte and mixed-analyte conditions. Table S1: Comparison of the charge transfer resistance (Rct) of Bare CC, ZnO/CC, V_2_O_5_/CC and ZVO/CC electrodes. Table S2: Quantitative summary of pre-reduction conditions, V^4+^ fraction, oxygen vacancy level, EPR response, charge transfer resistance (Rct), and sensing current. Table S3: Comparison of thiophanate-methyl detection performance between this work and previously reported methods. Table S4: Comparison of diuron detection performance between this work and previously reported methods.

## References

[1] Damalas C.A. (2018). Pesticides in agriculture: Environmental and health risks. Curr. Opin. Environ. Sci. Health.

[2] Lushchak V.I., Matviishyn T.M., Husak V.V., Storey J.M., Storey K.B. (2018). Pesticide toxicity: A mechanistic approach. EXCLI J..

[3] Deosthali C., Falaque T., Varjani S. (2026). Addressing ecological and human health impacts of pesticides and their remediation using integrated approaches. Int. Biodeterior. Biodegrad..

[4] Paul K.C., Krolewski R.C., Lucumi Moreno E., Blank J., Holton K.M., Ahfeldt T., Furlong M., Yu Y., Cockburn M., Thompson L.K. (2023). A pesticide and iPSC dopaminergic neuron screen identifies and classifies Parkinson-relevant pesticides. Nat. Commun..

[5] Mostafalou S., Abdollahi M. (2017). Pesticides: An update of human exposure and toxicity. Arch. Toxicol..

[6] Umapathi R., Ghoreishian S.M., Sonwal S., Rani G.M., Huh Y.S. (2022). Portable electrochemical sensing methodologies for on-site detection of pesticide residues in fruits and vegetables. Coord. Chem. Rev..

[7] Umapathi R., Rani G.M., Kim E., Park S.Y., Cho Y., Huh Y.S. (2022). Sowing kernels for food safety: Importance of rapid on-site detction of pesticide residues in agricultural foods. Food Front..

[8] Dixit A., Kumar N., Upadhyay A., Kaushik N. (2026). Nanotechnology in the detoxification of agrochemical residues. Nanotechnology in Pre- and Postharvest Horticulture.

[9] Durak B.Y., Chormey D.S., Firat M., Bakirdere S. (2020). Validation of ultrasonic-assisted switchable solvent liquid phase microextraction for trace determination of hormones and organochlorine pesticides by GC–MS and combination with QuEChERS. Food Chem..

[10] Liu J. (2020). High-sensitivity detection method for organochlorine pesticide residues based on loop-shaped absorber. Mater. Chem. Phys..

[11] Zhu A., Xu Y., Ali S., Ouyang Q., Chen Q. (2021). Au@Ag nanoflowers based SERS coupled chemometric algorithms for determination of organochlorine pesticides in milk. LWT.

[12] Mirres A.C.d.M., Silva B.E.P.d.M.d., Tessaro L., Galvan D., Andrade J.C.d., Aquino A., Joshi N., Conte-Junior C.A. (2022). Recent advances in nanomaterial-based biosensors for pesticide detection in foods. Biosensors.

[13] Maheshwaran S., Chen W.-H., Lin S.-L., Ghorbani M., Hoang A.T. (2024). Metal oxide-based electrochemical sensors for pesticide detection in water and food samples: A review. Environ. Sci. Adv..

[14] Han Y., Zhu L., Wen R., Liao J., Li B., Wu L. (2025). Innovative electrochemical sensors for pesticide residue detection: Nanomaterials, miniaturization, and intelligent data analysis. Chem. Rec..

[15] Jonatas de Oliveira S.S., Dos Santos J.F., Granja H.S., Almeida W.S., Loeser T.F., Freitas L.S., Bergamini M.F., Marcolino-Junior L.H., Sussuchi E.M. (2024). Simultaneous determination of carbendazim and carbaryl pesticides in water bodies samples using a new voltammetric sensor based on Moringa oleifera biochar. Chemosphere.

[16] Wang K., Cao Z., Ding Q., Yoo J., Singh N., Kang H., Wang L., Xu L., Kim J.S. (2024). Electronic structure tuning of layered double hydroxides for electrochemical sensing and its biomedical applications. Sci. China Chem..

[17] Çelik M., Silah H., Uslu B. (2025). Nanosheets based electrochemical sensors for pesticides detections in food and environmental matrices. Food Chem..

[18] Song D., Xu X., Huang X., Li G., Zhao Y., Gao F. (2023). Oriented design of transition-metal-oxide hollow multishelled micropolyhedron derived from bimetal–organic frameworks for the electrochemical detection of multipesticide residues. J. Agric. Food Chem..

[19] Yang J., Deng C., Zhong W., Peng G., Zou J., Lu Y., Gao Y., Li M., Zhang S., Lu L. (2023). Electrochemical activation of oxygen vacancy-rich TiO_2_@MXene as high-performance electrochemical sensing platform for detecting imidacloprid in fruits and vegetables. Microchim. Acta.

[20] Gong J., Miao X., Wan H., Song D. (2012). Facile synthesis of zirconia nanoparticles-decorated graphene hybrid nanosheets for an enzymeless methyl parathion sensor. Sens. Actuators B Chem..

[21] Karuppusamy N., Sakthivel R., Chen S.-M., Prasanna S.B., Chung R.-J. (2023). Template-assisted synthesis of Co_1_Zn_0.5_–P/CoO/ZnO core-shell heterostructure for the electrochemical detection of carbofuran and diuron. Chem. Eng. J..

[22] Ozcelikay G., Karadurmus L., Bilge S., Sınağ A., Ozkan S.A. (2022). New analytical strategies amplified with 2D carbon nanomaterials for electrochemical sensing of food pollutants in water and soils sources. Chemosphere.

[23] Tümay S.O., Şenocak A., Sarı E., Şanko V., Durmuş M., Demirbas E. (2021). A new perspective for electrochemical determination of parathion and chlorantraniliprole pesticides via carbon nanotube-based thiophene-ferrocene appended hybrid nanosensor. Sens. Actuators B Chem..

[24] Gunkel F., Christensen D.V., Chen Y., Pryds N. (2020). Oxygen vacancies: The (in)visible friend of oxide electronics. Appl. Phys. Lett..

[25] Sohrabi H., Arbabzadeh O., Falaki M., Majidi M.R., Han N., Yoon Y., Khataee A. (2022). Electrochemical layered double hydroxide (LDH)-based biosensors for pesticides detection in food and environment samples: A review of status and prospects. Food Chem. Toxicol..

[26] Zhou Q., Lin K., Liu Y., Sun G., Wang S., Zhai H. (2024). Determination of methyl parathion and fenitrothion on a Mg-Al layered double hydroxide/poly-m-amino-benzenesulfonic acid film-coated glassy carbon electrode (Mg-Al LDH/pm-ABSA/GCE). Electrochim. Acta.

[27] Zavalij P.Y., Zhang F., Whittingham M.S. (1997). A new zinc pyrovanadate, Zn_3_(OH)_2_V_2_O_7_.2H_2_O, from X-ray powder data. Cryst. Struct. Commun..

[28] Li Y., Teng Y., Zhang Z., Feng Y., Xue P., Tong W., Liu X. (2017). Microwave-assisted synthesis of novel nanostructured Zn_3_(OH)_2_V_2_O_7_·2H_2_O and Zn_2_V_2_O_7_ as electrode materials for supercapacitors. New J. Chem..

[29] Badawy M.E., El-Nouby M.A., Marei A.E.-S.M. (2018). Development of a Solid-Phase Extraction (SPE) Cartridge Based on Chitosan-Metal Oxide Nanoparticles (Ch-MO NPs) for Extraction of Pesticides from Water and Determination by HPLC. Int. J. Anal. Chem..

[30] Meephon S., Rungrotmongkol T., Puttamat S., Praserthdam S., Pavarajarn V. (2019). Heterogeneous photocatalytic degradation of diuron on zinc oxide: Influence of surface-dependent adsorption on kinetics, degradation pathway, and toxicity of intermediates. J. Environ. Sci..

[31] Ouedraogo B., Tall A., Bako Y.F.R., Tapsoba I. (2023). Sensitive determination of diuron on zinc oxide nanoparticles modified carbon paste electrode in soil and water samples. Electroanalysis.

[32] Zhang J., Jiang H., Gao J., Zhao C., Suo H. (2025). Three-dimensional CeO_2_ Nanosheets/CuO nanoflowers pn heterostructure supported on carbon cloth as electrochemical sensor for sensitive nitrite detection. Anal. Chim. Acta.

[33] Fallatah A., Kuperus N., Almomtan M., Padalkar S. (2022). Sensitive biosensor based on shape-controlled ZnO nanostructures grown on flexible porous substrate for pesticide detection. Sensors.

[34] Ganesan M., Devi R.K., Chen S.-M., Ravi S.K. (2023). Sustainable synthesis of hierarchical dysprosium vanadate 3D-micro flowers for electrochemical evaluation of organophosphate pesticide in food samples. Chem. Eng. J..

[35] Maheshwaran S., Govindharaj A., Chen S.-M., Tamilalagan E., Liu T.-Y. (2025). Emerging insights into dysprosium vanadate wrapped graphene oxide nanocomposites for electrochemical detection of carbendazim in water and food samples. Food Chem..

[36] Lee K., Ha S.M., Gurudatt N., Heo W., Hyun K.-A., Kim J., Jung H.-I. (2024). Machine learning-powered electrochemical aptasensor for simultaneous monitoring of di (2-ethylhexyl) phthalate and bisphenol A in variable pH environments. J. Hazard. Mater..

[37] Kordek K., Jiang L., Fan K., Zhu Z., Xu L., Al-Mamun M., Dou Y., Chen S., Liu P., Yin H. (2019). Two-step activated carbon cloth with oxygen-rich functional groups as a high-performance additive-free air electrode for flexible zinc–air batteries. Adv. Energy Mater..

[38] Zhao X., Kou X., Li X., Li M., Xi D., Farid A., Huang H., Zhao Y. (2025). N-doped carbon nanotubes as electron transport promoter by bridging MnFe_2_O_4_ and carbon cloth for simultaneous detection of heavy metal ions (Cd^2+^ and Pb^2+^). Microchem. J..

[39] Ren J., Hong P., Ran Y., Wang B., Chen T., Wang Y. (2022). High-loading and high-performance zinc ion batteries enabled by electrochemical conversion of vanadium oxide cathodes. Electrochim. Acta.

[40] Kokulnathan T., Wang T.-J., Murugesan T., Anthuvan A.J., Kumar R.R., Ahmed F., Arshi N. (2023). Structural growth of zinc oxide nanograins on carbon cloth as flexible electrochemical platform for hydroxychloroquine detection. Chemosphere.

[41] Yang G., Li S., Wu M., Wang C. (2016). Zinc pyrovanadate nanosheets of atomic thickness: Excellent Li-storage properties and investigation of their electrochemical mechanism. J. Mater. Chem. A.

[42] Popov A., Kotomin E., Maier J. (2010). Basic properties of the F-type centers in halides, oxides and perovskites. Nucl. Instrum. Methods Phys. Res. Sect. B Beam Interact. Mater. At..

[43] Chaiyo S., Mehmeti E., Žagar K., Siangproh W., Chailapakul O., Kalcher K. (2016). Electrochemical sensors for the simultaneous determination of zinc, cadmium and lead using a Nafion/ionic liquid/graphene composite modified screen-printed carbon electrode. Anal. Chim. Acta.

[44] Choi Y.-H. (2022). VO2 as a highly efficient electrocatalyst for the oxygen evolution reaction. Nanomaterials.

[45] Gurusamy L., Karuppasamy L., Anandan S., Barton S.C., Chuang Y.-H., Liu C.-H., Wu J.J. (2023). Review of oxygen-vacancies nanomaterials for non-enzymatic electrochemical sensors application. Coord. Chem. Rev..

[46] Singh S.B., Foster G.D., Khan S.U. (2007). Determination of thiophanate methyl and carbendazim residues in vegetable samples using microwave-assisted extraction. J. Chromatogr. A.

[47] Jiang J., Li S., Yan Y., Fang M., Chen M., Peng K., Nie L., Feng Y., Wang X. (2017). Pendant structure governed the selectivity of Pd^2+^ using disubstituted polyacetylenes with sulfur functions and the application of thiophanate-methyl detection. Sens. Actuators B Chem..

[48] Nguyen Thi Nhat H., Le N.T.T., Thi Phuong Phong N., Nguyen D.H., Nguyen-Le M.-T. (2020). Hydroquinone-based fabrication of gold nanorods with a high aspect ratio and LSPR greater than 850 nm to be used as a surface plasmon resonance platform for rapid detection of thiophanate methyl. Appl. Sci..

[49] Zheng M., Wang Y., Wang C., Wei W., Ma S., Sun X., He J. (2018). Silver nanoparticles-based colorimetric array for the detection of Thiophanate-methyl. Spectrochim. Acta Part A Mol. Biomol. Spectrosc..

[50] Yue X., Zhu C., Gu R., Hu J., Xu Y., Ye S., Zhu J. (2022). Copper-modified double-emission carbon dots for rapid detection of thiophanate methyl in food. Foods.

[51] Fu Y., Yang T., Zhao J., Zhang L., Chen R., Wu Y. (2017). Determination of eight pesticides in Lycium barbarum by LC-MS/MS and dietary risk assessment. Food Chem..

[52] Wei F., Wu J., Luo Y., Xu H., Feng D., Huang K., Tan X. (2025). Synergistic electrochemical-colorimetric dual-mode sensor for thiophanate-methyl detection in water samples using bimetallic porphyrin MOF nanosheets. Microchem. J..

[53] Li Z., Wang M., Zhang Y., Bian Z., Liu X., Bai L., Liu A. (2025). Biofuel cell assembled by Cu_2_O nanocube laccase-mimicking nanozyme based sensitive self-powered sensor to detect fungicide thiophanate-methyl. Microchem. J..

[54] Oliveira R.S., da Silva H.B., de Souza C.C., Veríssimo W.B., Matos M.A.C., Lisboa T.P., Matos R.C. (2024). Development of an electrochemical sensor utilizing recycled ABS filaments for 3D printing in the determination of diuron. Microchem. J..

[55] Duarte E.H., Casarin J., Sartori E.R., Tarley C.R.T. (2018). Highly improved simultaneous herbicides determination in water samples by differential pulse voltammetry using boron-doped diamond electrode and solid phase extraction on cross-linked poly (vinylimidazole). Sens. Actuators B Chem..

[56] Wong A., Foguel M.V., Khan S., de Oliveira F.M., Tarley C.R.T., Sotomayor M.D. (2015). Development of an electrochemical sensor modified with MWCNT-COOH and MIP for detection of diuron. Electrochim. Acta.

[57] de Matos Morawski F., Winiarski J.P., de Campos C.E.M., Parize A.L., Jost C.L. (2020). Sensitive simultaneous voltammetric determination of the herbicides diuron and isoproturon at a platinum/chitosan bio-based sensing platform. Ecotoxicol. Environ. Saf..

[58] Zhu J., He Y., Luo L., Li L., You T. (2023). Electrochemical determination of hazardous herbicide diuron using MWCNTs-CS@NGQDs composite-modified glassy carbon electrodes. Biosensors.

[59] Asgari S., Wu G., Aghvami S.A., Zhang Y., Lin M. (2021). Optimisation using the finite element method of a filter-based microfluidic SERS sensor for detection of multiple pesticides in strawberry. Food Addit. Contam. Part A.

[60] Puthongkham P., Wirojsaengthong S., Suea-Ngam A. (2021). Machine learning and chemometrics for electrochemical sensors: Moving forward to the future of analytical chemistry. Analyst.

[61] Akarsu C.H., Küçükdeniz T., Tüzün E., Karakuş S. (2026). ElectroML: An open-source web platform for machine learning-based analyte concentration prediction in electrochemical sensing. SoftwareX.

