# Supplementary material for: Pre-Reduction-Activated Carbon-Based Zinc Vanadate Nanosheet Composite with Zn–O–V Performance Synergy for Machine Learning-Enabled Multiplex Pesticide Detection
Source: Nanomaterials (Basel). 2026 Aug 31;16(17):1082. doi: 10.3390/nano16171082 (PMC13567297; doi:10.3390/nano16171082)
Supplement: Supplementary file 1 [file nanomaterials-16-01082-s001.zip › nanomaterials-4530120-supplementary.pdf]

Supplementary Materials

Article

Pre-Reduction-Activated Carbon-Based Zinc Vanadate Nanosheet Composite with Zn–O–V Performance Synergy for Machine Learning-Enabled Multiplex Pesticide Detection

Lihua Zhong [1,†], Bingrui Zou [2,†], Xin Li [1], Shuiju Guo [3], Haijun Guan [1], Chou Mo [1], Qianfeng Wang [1], Yuchao Wang [1], Hongyu Wang [4,*], Xin Kou [5,*], Yongpeng Zhao [1,*] and Hui Huang [1,*]

[1] College of Mechanical and Electrical Engineering, Sichuan Agricultural University, Ya'an 625000, China
[2] College of Water Conservancy and Hydropower Engineering, Sichuan Agricultural University, Ya'an 625000, China
[3] Party School of CPC Wuwei Municipal Committee (Wuwei Administrative College), Wuwei 733000, China
[4] College of Mechanical Engineering, Qinghai University, Xining 810016, China
[5] College of Resources, Sichuan Agricultural University, Chengdu 610000, China
[*] Correspondence: hyuwang26@163.com (H.W.); 14910@sicau.edu.cn (X.K.); zhaoyp@sicau.edu.cn (Y.Z.); huanghui@sicau.edu.cn (H.H.)
[†] These authors contribute equally to this work.

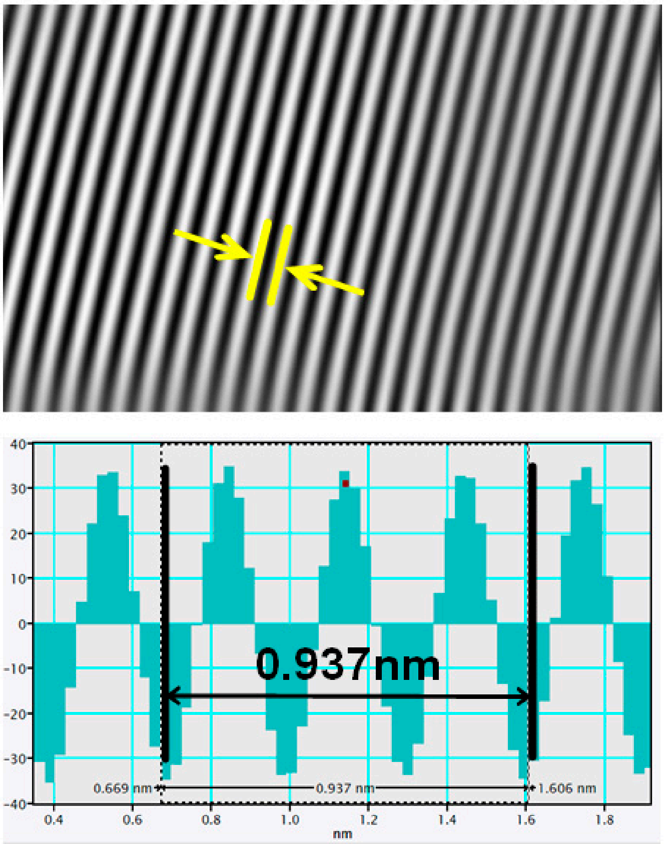

Figure S1. Interplanar spacing analysis of ZVO.

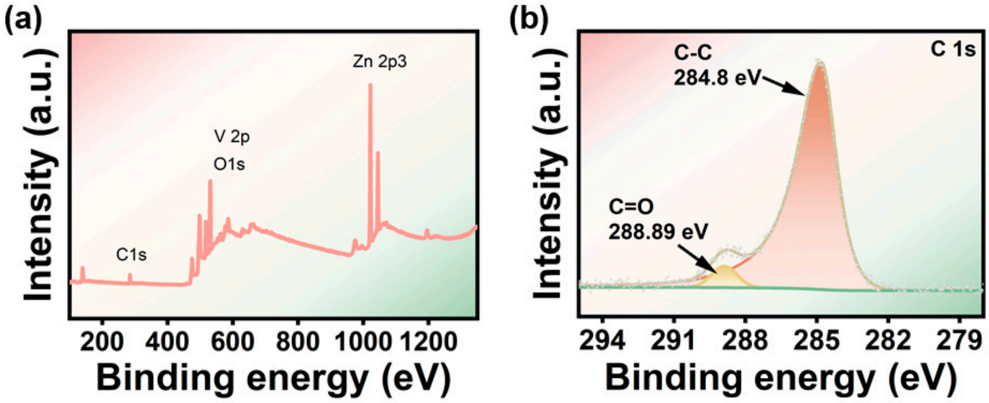

Figure S2. (a) XPS survey spectrum of ZVO/CC. (b) High-resolution C 1s spectrum.

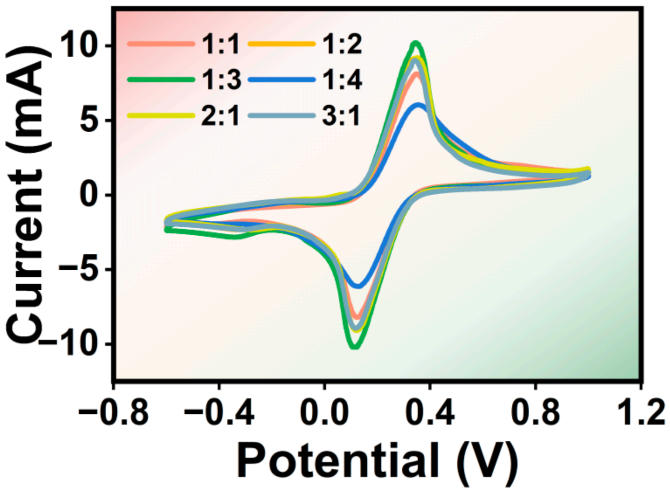

Figure S3. CV curves of ZVO/CC prepared with different precursor Zn:V ratios.

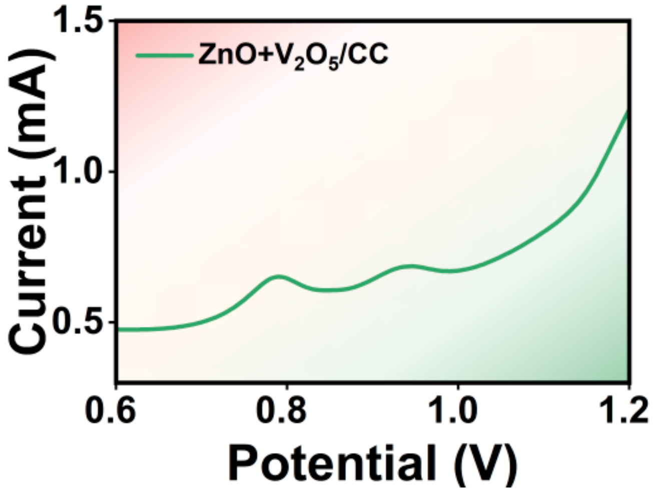

Figure S4. Electrochemical sensing toward thiophanate-methyl and diuron for ZnO+V_2O_5/CC.

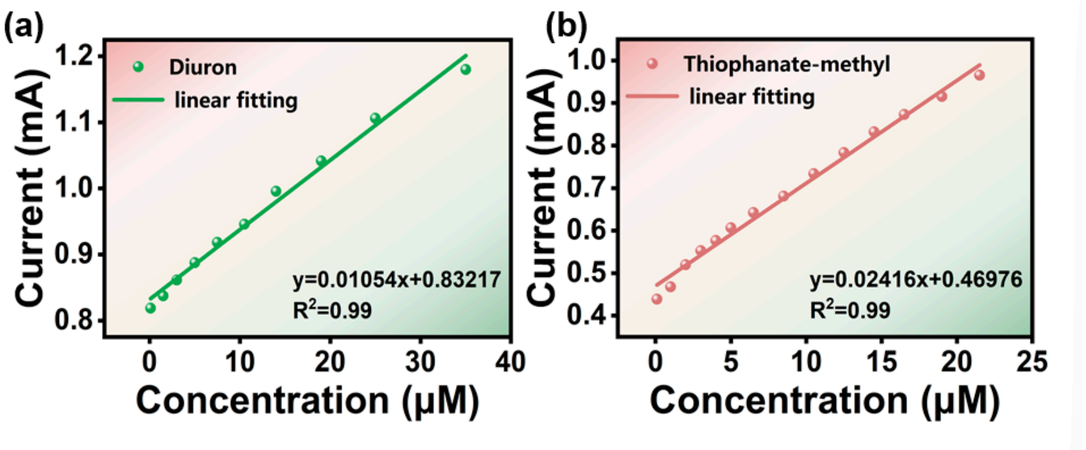

Figure S5. Linear relationships between analyte concentration and oxidation peak current for (a) thiophanate-methyl and (b) diuron, respectively. thiophanate-methyl 14 points, diuron 10 points, n=3 per concentration, blank n=5.

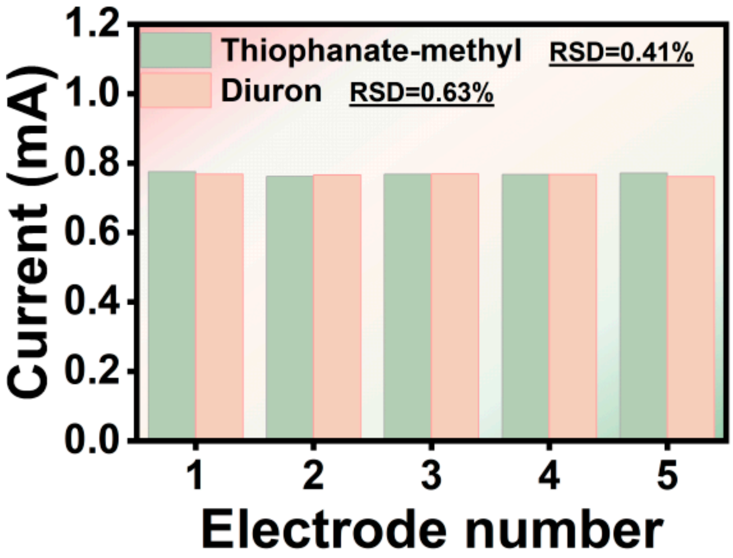

Figure S6. Electrode-to-electrode reproducibility of independently prepared ZVO/CC electrodes for the detection of thiophanate-methyl and diuron.

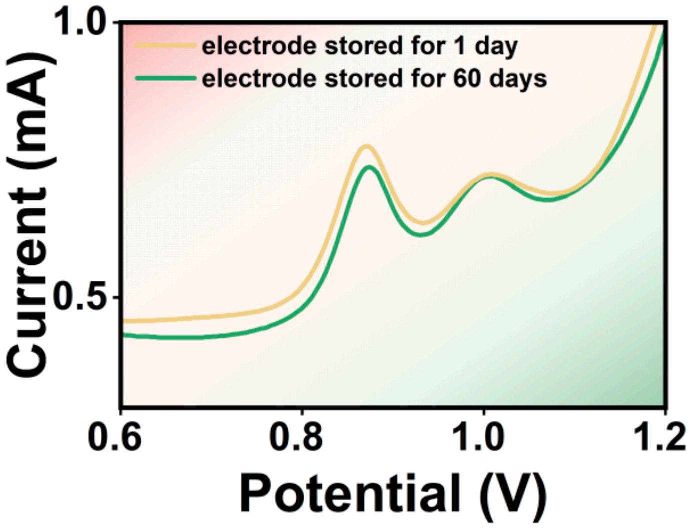

Figure S7. Long-term storage stability of the ZVO/CC electrode toward thiophanate-methyl and diuron after sealed storage at room temperature for 60 days.

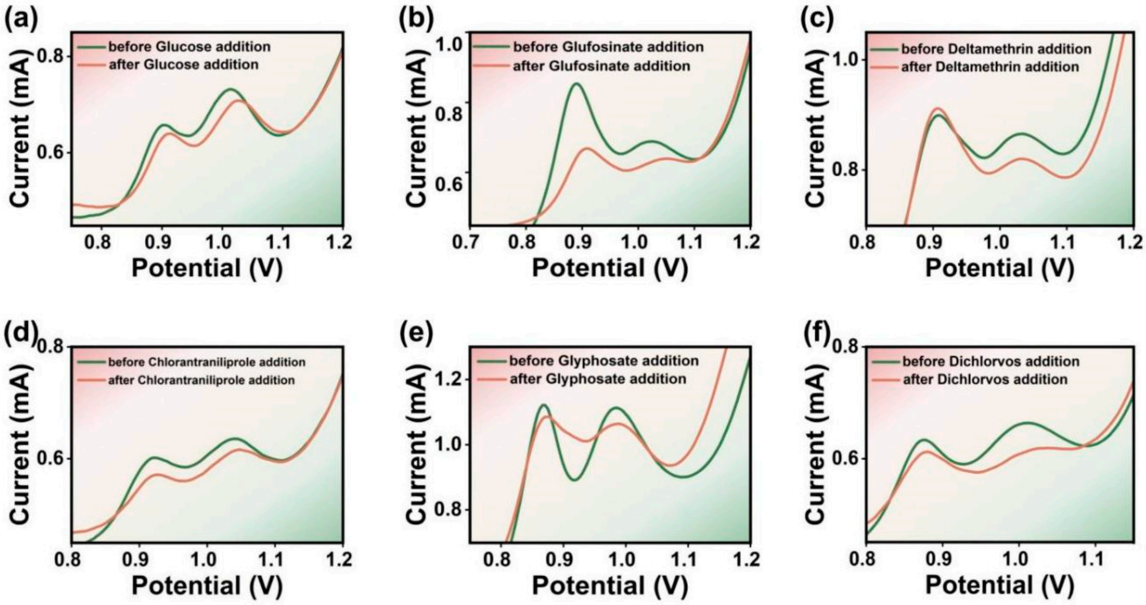

Figure S8. DPV responses of the ZVO/CC electrode toward thiophanate-methyl and diuron before and after the addition of fivefold concentrations of (a) glucose, (b) glufosinate, (c) deltamethrin, (d) chlorantraniliprole, (e) glyphosate, and (f) dichlorvos.

class	precision	recall	f1-score	support
Diuron	1.00	0.94	0.97	71
Mixture	0.90	0.99	0.94	71
None	0.96	1.00	0.98	71
Thiophanate-methyl	0.98	0.90	0.94	71
accuracy	None	None	0.96	284
macro avg	0.96	0.96	0.96	284
weighted avg	0.96	0.96	0.96	284

Figure S9. Class-wise and overall performance metrics of the SVM classifier for pesticide discrimination.

sample type	target analyte	Train R^2	Test R^2	RMSE (µM)	MAE (µM)
**Single-analyte**	**Thiophanate-methyl**	0.989	0.966	2.027	1.419
**Single-analyte**	**Diuron**	0.993	0.949	2.235	1.554
**Mixture**	**Thiophanate-methyl**	0.965	0.900	1.253	0.832
**Mixture**	**Diuron**	0.964	0.895	1.861	1.234

Figure S10. Quantitative performance metrics of the random-forest concentration-prediction model for thiophanate-methyl and diuron under single-analyte and mixed-analyte conditions.

Table S1. Comparison of the charge transfer resistance (Rct) of Bare CC, ZnO/CC, V_2O_5/CC and ZVO/CC electrodes.

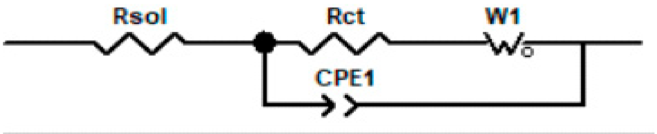

Electrode	Charge transfer resistance (Rct, ohm)
Bare CC	4.654
ZnO/CC	4.261
V_2O_5/CC	3.432
ZVO/CC	3.378

Table S2. Quantitative summary of pre-reduction conditions, V^{4+} fraction, oxygen vacancy level, EPR response, charge transfer resistance (Rct), and sensing current.

Electrode	Pre-reduction condition	V 2p spectral evolution	EPR response	Rct (Ω)	Thiophanate-methyl response	Diuron response
**ZVO/CC**	Without pre-reduction	Only V^{5+}	Lower EPR intensity	3.378	No discernible oxidation peak	No discernible oxidation peak
**Pre-reduced ZVO/CC**	−1.0 to 0 V	Coexistence of V^{4+} and V^{5+}	Enhanced EPR intensity	NA	Enhanced oxidation peak at +0.86 V	Enhanced oxidation peak at +1.00 V

Table S3. Comparison of thiophanate-methyl detection performance between this work and previously reported methods

Analytical technique	Linear Range (μM)	LOD (μM)	Matrix	No. of analytes	Simultaneous detection	Ref.
HPLC	1.6-11.68	0.15	NR	1	No	[1]
Fluorescence	0-4	0.012	NR	1	No	[2]
SERS (AuNRs)	2.92-20.44	14.5	NR	1	No	[3]
UV-vis Spectrometry	2-100	0.12	NR	1	No	[4]
Fluorescent sensor (Cu-CDs)	0-0.65	0.75 (peak A)<br>0.78 (peak B)	Food samples	1	No	[5]
LC-MS/MS	0.23-0.53	61.3	Lycium barbarum fruits	8	No	[6]
EC/Color (PMOF/MXene)	0.05-1.7	0.0101<br>0.0311	Water samples	1	No	[7]
Cu_2O NCs/GCE	0.5-40	0.11	NR	1	No	[8]

HPLC: High performance liquid chromatography.

Fluorescence: Fluorescence spectrometry.

SERS (AuNRs): Surface enhanced Raman spectroscopy using gold nanorods.

UV-vis Spectrometry: Ultraviolet visible absorption spectrometry.

Fluorescent sensor (Cu-CDs): Fluorescent sensor based on copper doped carbon dots.

LC-MS/MS: Liquid chromatography tandem mass spectrometry.

EC/Color (PMOF/MXene): Electrochemical and colorimetric method using porphyrinic metal organic framework combined with MXene composite.

Cu_2O NCs/GCE: Copper(I) oxide nanocubes modified glassy carbon electrode.

Table S4. Comparison of diuron detection performance between this work and previously reported methods

Electrode	Linear Range(μM)	LOD (μM)	Matrix	No. of analytes	Simultaneous detection	Ref.
3D-CPE	0.25-20.0	0.07	Sugar cane juice; cachaça	1	No	[9]
BDDE	1.0-9.0	0.035	Lake water; well water	3	Yes	[10]
MWCNT-COOH-MIP/CPE	0.05-1.25	0.009	River water	1	No	[11]
PtNPs/CS/ GCE	0.17-4.29	0.0858	River water	2	Yes	[12]
MWCNTs-CS@NGQDs/GCE	0.08-12.0	0.04	River water; soil	1	No	[13]

3D-CPE: Three dimensional carbon paste electrode.

BDDE: Boron doped diamond electrode.

MWCNT-COOH-MIP/CPE: Carboxyl functionalized multi walled carbon nanotube molecularly imprinted polymer combined with carbon paste electrode.

PtNPs/CS/GCE: Platinum nanoparticles combined with chitosan modified glassy carbon electrode.

MWCNTs-CS@NGQDs/GCE: Multi walled carbon nanotubes combined with chitosan loaded with nitrogen doped graphene quantum dots modified glassy carbon electrode.

References

[1] S.B. Singh, G.D. Foster, S.U. Khan, Determination of thiophanate methyl and carbendazim residues in vegetable samples using microwave-assisted extraction, Journal of Chromatography A 1148(2), 152-157 (2007).

[2] J. Jiang, S. Li, Y. Yan, et al., Pendant structure governed the selectivity of Pd2+ using disubstituted polyacetylenes with sulfur functions and the application of thiophanate-methyl detection, Sensors and Actuators B: Chemical 247, 36-45 (2017).

[3] H. Nguyen Thi Nhat, N.T.T. Le, N. Thi Phuong Phong, et al., Hydroquinone-based fabrication of gold nanorods with a high aspect ratio and LSPR greater than 850 nm to be used as a surface plasmon resonance platform for rapid detection of thiophanate methyl, Applied Sciences 10(10), 3654 (2020).

[4] M. Zheng, Y. Wang, C. Wang, et al., Silver nanoparticles-based colorimetric array for the detection of Thiophanate-methyl, Spectrochimica Acta Part A: Molecular and Biomolecular Spectroscopy 198, 315-321 (2018).

[5] X. Yue, C. Zhu, R. Gu, et al., Copper-modified double-emission carbon dots for rapid detection of thiophanate methyl in food, Foods 11(21), 3336 (2022).

[6] Y. Fu, T. Yang, J. Zhao, et al., Determination of eight pesticides in Lycium barbarum by LC-MS/MS and dietary risk assessment, Food Chemistry 218, 192-198 (2017).

[7] F. Wei, J. Wu, Y. Luo, et al., Synergistic electrochemical-colorimetric dual-mode sensor for thiophanate-methyl detection in water samples using bimetallic porphyrin MOF nanosheets, Microchemical Journal 220, 116465 (2026).

[8] Z. Li, M. Wang, Y. Zhang, et al., Biofuel cell assembled by Cu2O nanocube laccase-mimicking nanozyme based sensitive self-powered sensor to detect fungicide thiophanate-methyl, Microchemical Journal 216, 114676 (2025).
[9] R.S. Oliveira, H.B. da Silva, C.C. de Souza, et al., Development of an electrochemical sensor utilizing recycled ABS filaments for 3D printing in the determination of diuron, Microchemical Journal 201, 110454 (2024).
[10] E.H. Duarte, J. Casarin, E.R. Sartori, et al., Highly improved simultaneous herbicides determination in water samples by differential pulse voltammetry using boron-doped diamond electrode and solid phase extraction on cross-linked poly (vinylimidazole), Sensors and Actuators B: Chemical 255, 166-175 (2018).
[11] A. Wong, M.V. Foguel, S. Khan, et al., Development of an electrochemical sensor modified with MWCNT-COOH and MIP for detection of diuron, Electrochimica Acta 182, 122-130 (2015).
[12] F.d.M. Morawski, J.P. Winiarski, C.E.M. de Campos, et al., Sensitive simultaneous voltammetric determination of the herbicides diuron and isoproturon at a platinum/chitosan bio-based sensing platform, Ecotoxicology and Environmental Safety 206, 111181 (2020).
[13] J. Zhu, Y. He, L. Luo, et al., Electrochemical determination of hazardous herbicide diuron using MWCNTs-CS@ NGQDs composite-modified glassy carbon electrodes, Biosensors 13(8), 808 (2023).
